# Hierarchical Microcarriers Loaded with Peptide Dendrimer‐Grafted Methotrexate for Rheumatoid Arthritis Treatment

**DOI:** 10.1002/smsc.202300097

**Published:** 2023-11-27

**Authors:** Yang Li, Haofang Zhu, Rui Liu, Yuanjin Zhao, Lingyun Sun

**Affiliations:** ^1^ Department of Rheumatology and Immunology Institute of Translational Medicine Nanjing Drum Tower Hospital Clinical Medical College of Traditional Chinese and Western Medicine Nanjing University of Chinese Medicine Nanjing 210096 China; ^2^ State Key Laboratory of Bioelectronics School of Biological Science and Medical Engineering Southeast University Nanjing 210096 China

**Keywords:** hydrogels, methotrexate, microcarriers, microfluidics, peptide dendrimers, rheumatoid arthritis

## Abstract

Rheumatoid arthritis (RA) is one of the leading causes of disability due to the autoimmune destruction of synovial joints. Great efforts have been put into developing multifunctional drug delivery systems for delaying the progression of RA. Herein, a novel locally injectable, hierarchically structured delivery system with a hyaluronic acid (HA) microparticle and an MTX‐conjugated nanoparticle for promoted RA treatment is proposed. In this system, MTX is chemically grafted onto a peptide dendrimer‐based nanoparticle (G‐MTX). These nanoparticles are encapsulated inside HA microparticles (H‐G‐MTX) using microfluidics to realize the system. The G‐MTX can be released from the degradable HA microparticles in the acidic environment of inflamed RA joints. As the G‐MTX shows an enhanced intracellular delivery of the conjugated MTX, the H‐G‐MTX can reverse the dominant macrophage phenotype from M1 to M2. Through a collagen‐induced arthritis (CIA) rat model, the great therapeutic outcomes of this hierarchically structured delivery system are demonstrated, including inflammation reduction, cartilage protection, and bone mineral density promotion. These results indicate that the proposed hierarchical delivery system has potential clinical value in the treatment of RA and other diseases.

## Graphical abstract

A novel locally injectable, hierarchically structured delivery system with hyaluronic acid particles and a methotrexate conjugated nanoparticle which enables precise accumulation, responsive release, and amplifies efficacy of methotrexate and target therapy in rheumatoid arthritis (RA) joints is studied. The system‐alleviated bone destruction and cartilage damage and these results indicate the potential of this system for RA treatment.

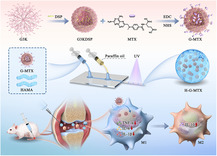

## 1 Introduction

Rheumatoid arthritis (RA) is a chronic systemic autoimmune disease, characterized by progressive joint destruction, bone erosion, synovial hyperplasia, and severe joint pain.^[^ ^1^, ^2^, ^3^, ^4^, ^5^, ^6^, ^7^, ^8^ ^]^ Thus, there is a long‐term therapeutic need for patients with RA. Methotrexate (MTX), a chemotherapeutic drug with anti‐inflammatory and immunoregulatory properties, is the first‐line drug and gold standard for the treatment of RA.^[^ ^3^, ^9^, ^10^, ^11^ ^]^ Whereas, the widely adopted systemic oral or intravenous administration of MTX drugs always demonstrated inevitable short‐acting and side effects. Through localized delivery, therapeutic drugs can be transported and concentrated precisely to the affected site.^[^ ^3^, ^5^, ^12^, ^13^, ^14^, ^15^, ^16^, ^17^, ^18^, ^19^, ^20^ ^]^ Therefore, a variety of nano‐ and microparticle‐based drug delivery carriers have been developed to overcome these barriers. However, it is difficult for these nanoparticles to deliver drugs intracellularly to show effective therapeutic effects. As an alternative, nanoparticles can be internalized by cells through charge trapping. Despite many successes, they are rapidly metabolized in the inflammatory environment of the joints, have a short residence time, and repeated dosing severely reduces efficacy and increases adverse events (e.g., bleeding), joint infections, and systemic side effects. Consequently, developing new drug delivery systems that are capable of precisely accumulating in RA joints, responsively releasing, and amplifying the functional effect of embedded drugs are still eagerly expected.

**Figure 1:**
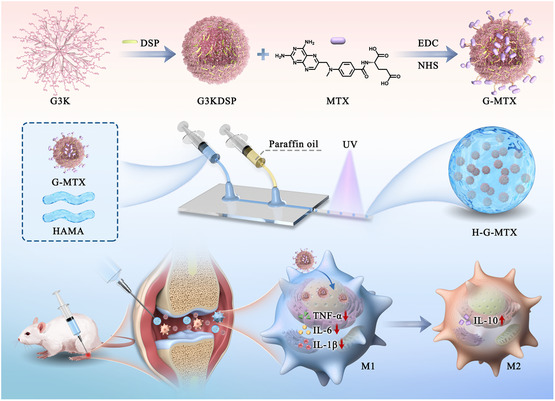
Schematic diagram of the locally injectable, hierarchical micro‐ and nanodelivery system with a HAMA microparticle and an MTX‐conjugated nanoparticle for RA treatment.

Here, inspired by the micro‐to‐nanohierarchical structure of the native cells, we proposed a novel locally injectable micro‐ and nanodelivery system for RA treatment, as schemed in **Figure** **1**. Compared to traditional linear polymers, peptide dendrimers are promising macromolecules with a well‐defined molecular weight, a highly branched architecture, a protein‐like globular structure, and substantial periphery groups. These unique properties make them ideal drug carriers by physical encapsulation or covalent conjugation.^[^ ^12^, ^17^, ^21^, ^22^, ^23^, ^24^ ^]^ Although the peptide dendrimer‐based nanoplatforms confer substantial benefits for drug delivery, including rapid cell entry and easier passage across biological barriers, their practical efficacy is still limited by uncontrolled targetability and stability. Recently, hyaluronic acid (HA) methacrylate (HAMA) hydrogel microcarriers prepared by microfluidic photocrosslinking technology were reported with uniform particle size monodisperse and excellent biocompatibility.^[^ ^25^, ^26^, ^27^, ^28^, ^29^, ^30^, ^31^, ^32^, ^33^ ^]^ They have superior injectability and can be minimally implanted into the joint cavity with sustained retention to reduce tissue damage and provide targeted treatment.^[^ ^16^, ^34^, ^35^, ^36^, ^37^, ^38^ ^]^ Thus, it is conceivable that the combination of peptide dendrimer‐based nanoparticles and HAMA microparticles as MTX carriers would provide a distinctive strategy for RA treatment.

In this article, we chemically grafted MTX onto the peptide dendrimer to form nanoparticles (G‐MTX). Using microfluidics, G‐MTX was encapsulated inside HAMA microspheres (H‐G‐MTX), enabling localized and hierarchical delivery of MTX, which could provide a unique strategy for the treatment of RA. By changing the feeding ratio of MTX, the nanoparticles with appropriate drug loading efficiency could be achieved. These nanoparticles were precisely loaded into monodisperse H‐G‐MTX microspheres with adjustable diameter and porous internal structure by varied microfluidic parameters. The resultant H‐G‐MTX showed reduced cytotoxicity and enhanced anti‐inflammatory effects against raw 264.7 macrophages. After being injected into an RA joint, where the environment is acidic, MTX could be sustainably released from the degradable HAMA microcarriers. It was demonstrated that the micro‐ and nanodelivery systems could considerably prolong the retention time of MTX in the inflamed joints. Importantly, the H‐G‐MTX exhibited desirable therapeutic outcomes in vivo, including reduced joint swelling, alleviated bone destruction, and less cartilage damage. These unique features suggest the value of a graded micro–nano delivery system in the treatment of RA and various autoimmune diseases.

## 2 Results and Discussion

**Figure 2:**
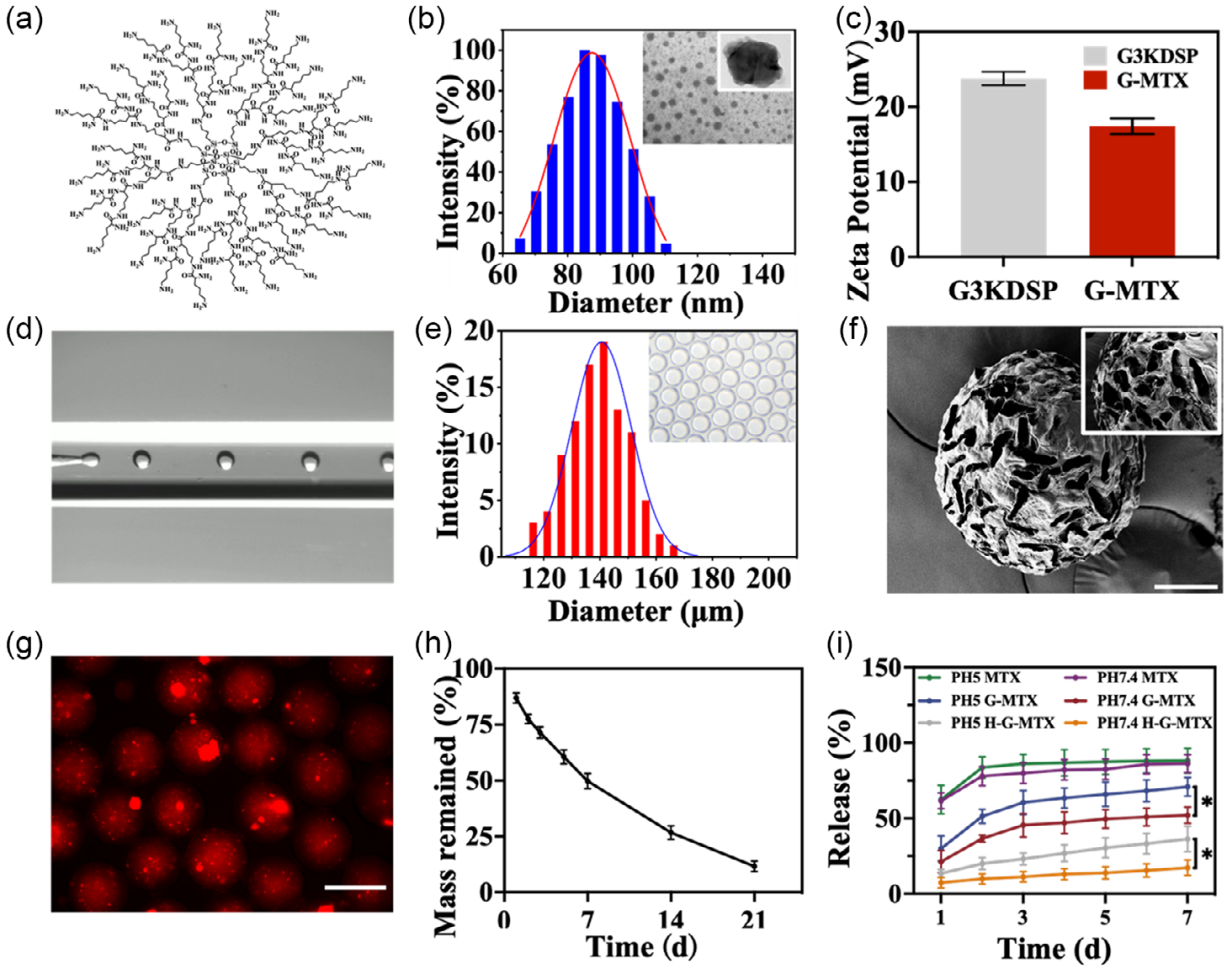
a) Peptide dendrimer structure. b) Size distribution of G‐MTX measured by DLS. c) Zeta Potential of dendrimers and dendrimers‐grafted MTX. d) Real‐time images of the H‐G‐MTX droplets in the microfluidic device. e) Particle size of HAMA microgels. f) TEM image of G‐MTX conjugated HAMA solution. Scale bar: 50 μm. g) CLSM image of H‐G‐MTX G‐MTX were labeled with AF750 NHS. Scale bar: 150 μm. h) Degradation curve of HAMA microspheres. i) Cumulative percent MTX released from free MTX, G‐MTX, and H‐G‐MTX at pH 5 and 7.4. n = 3. Data were presented as mean ± SD.

According to the classical divergent method previously reported,^[^ ^35^ ^]^ peptide dendrimer (G3K) based on polyhedral oligomeric silsesquioxane core was synthesized in high yield (**Figure** **2**a). G3K formed nanoparticles through propanoic acid,3,3′‐dithiobis‐,1,1′‐bis (2,5‐dioxo‐1‐pyrrolidinyl) ester (DSP) crosslinking (Figure S1, Supporting Information). To prepare drug‐loaded nanoparticles, nanoparticles were grafted with MTX in dimethyl sulfoxide (DMSO) in the proportions of 1:2, 1:5, and 1:10, respectively. According to ^1^H nuclear magnetic resonance (^1^H NMR) and ultraviolet and visible spectrophotometry (UV–vis) absorption spectroscopy, the encapsulation ratio, drug loading, and grafting ratio were the highest under the proportion of 1:10 (Figure S2, and S3a, Supporting Information). Finally, the polymers used in the subsequent experiments were chosen to be grafted at a 1:10 ratio, and the average particle size of the obtained nanoparticles was 85 ± 25 nm (Figure 2b). Grafted G‐MTX improves the water solubility of MTX and thus reduces the toxicity of MTX solubilization using organic reagents. As the time and dilution ratio increase, what remains constant is the size of the nanoparticles and the zeta potential, suggesting that nanoparticles had good stability (Figure S4, Supporting Information). After grafting with MTX, the zeta potential of the dendrimer changed a little and still carries massive positive charges, and due to electrostatic interactions, the cationic polymers can remove negatively charged inflammatory cytokines and cellular debris to impede the progression of arthritic inflammation in the RA model (Figure 2c).^[^ ^39^, ^40^, ^41^, ^42^ ^]^

To mimic the cellular hierarchical structure, the formulated G‐MTX was loaded into hydrogel microspheres (H‐G‐MTX) using microfluidics. HAMA was an ideal material for the preparation of hydrogel microspheres because of its unique biocompatibility and its ability to lubricate joints.^[^ ^37^ ^]^ The microfluidic device was composed of internal and external capillary glass tubes mounted coaxially in a square glass tube. The intermediate channel containing HAMA and nanoparticles is a dispersed phase, and the external channel containing liquid paraffin and span 80 is a continuous phase. The liquid paraffin exerts a shear force on the inner phase solution, causing the mixture of HAMA and nanoparticles to split into emulsion droplets (Figure 2d). The liquid droplets generated at the outlet are polymerized into microspheres under UV. By adjusting the two‐phase flow rate, HAMA‐G‐MTX microspheres with an average diameter of 145 ± 25 μm (Figure 2e) were chosen for further study. In addition, freeze‐dried HAMA microspheres were observed under scanning electron microscopy (SEM). Uniform voids in the microspheres can be observed (Figure 2f). To prove that drug‐loaded nanoparticles were successfully loaded in HAMA microspheres, Alexa Fluor® 750 NHS (AF750 NHS) Ester‐labeled nanoparticles were fabricated and the microspheres loaded with nanoparticles were observed under a fluorescence microscope. The nanoparticles loaded inside the microspheres show strong red fluorescence, while showing very weak fluorescence outside the microsphere (Figure 2g), suggesting that the nanoparticles were effectively immobilized in the microspheres network. The drug loading efficiency (10.52%) and encapsulation efficiency (73.6%) of the microspheres were calculated by UV–vis absorption spectroscopy to provide a reference for subsequent administration (Figure S3b, Supporting Information).

To simulate the gradual degradation process of microspheres in vitro, they were placed in phosphate‐buffered solution (PBS) containing hyaluronidase at 37 °C for 3 weeks. The quality of microspheres gradually decreased within 3 weeks. The mass of microspheres was reduced by nearly 90% after 21 days. This slow degradation was conducive to the long‐term release of drugs (Figure 2h). The nanoparticles loaded inside the microspheres show strong red fluorescence while showing very weak fluorescence outside the microsphere. The release mode of free MTX at pH 5 and pH 7.4 was similar (Figure 2i). A large number of drugs were released completely within 1 day. G‐MTX showed rapid release under acidic conditions. Within 4 days, drugs were completely released under pH 5, and nearly 80% were released under pH 7.4. Within 12 days, the drugs were completely released at pH 5 and nearly 90% at pH 7.4. The high release of cumulative drugs with pH 5 is of great value, especially in the inflammatory site, which would play a better therapeutic effect than free MTX.^[^ ^38^ ^]^ When G‐MTX was loaded into HAMA microspheres, the microspheres acted as an additional barrier to drug release, resulting in a significant slow and sustained release pattern of G‐MTX.

**Figure 3:**
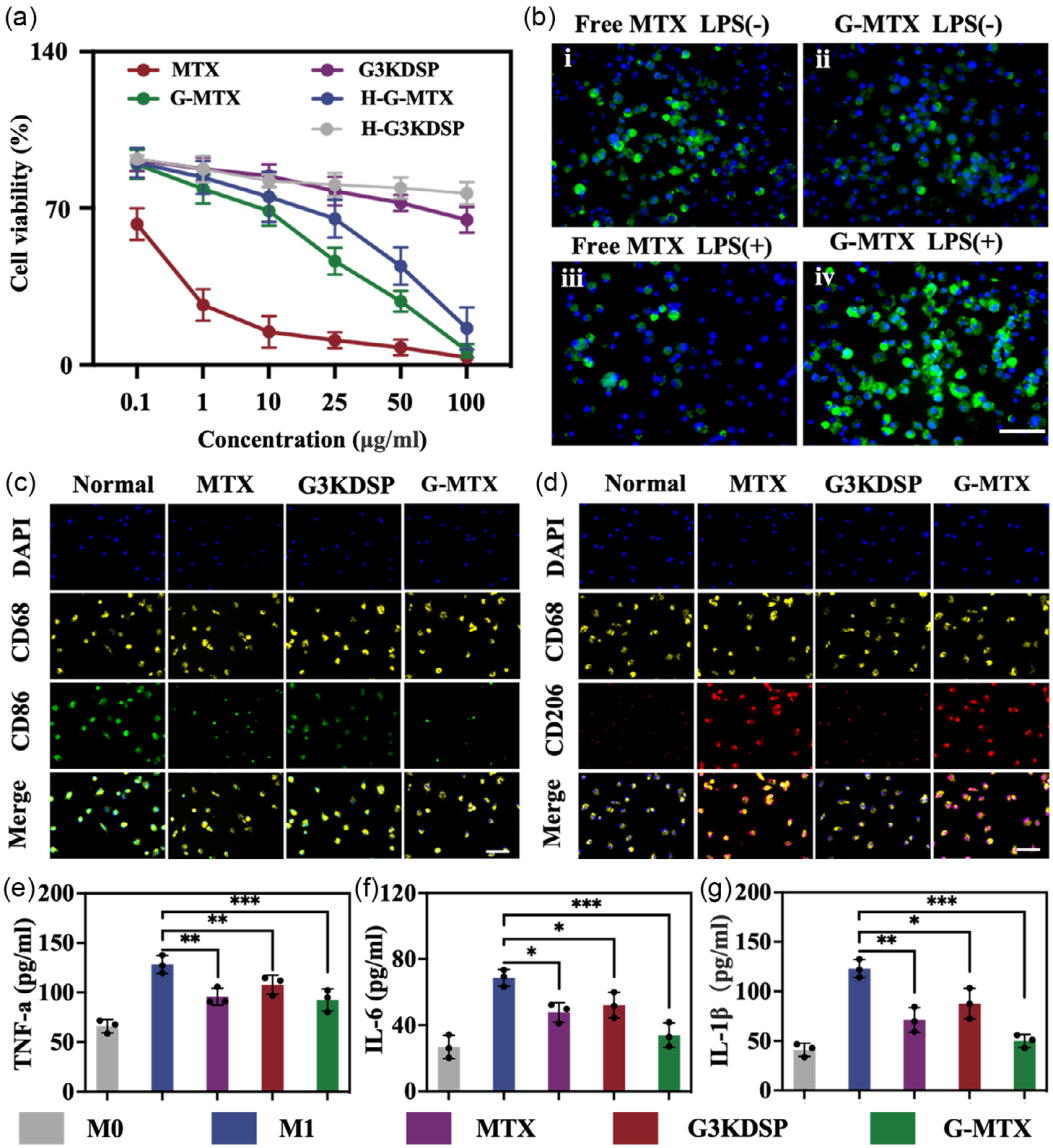
a) The cytotoxicity of G3KDSP, MTX, G‐MTX, H‐G3KDSP, or H‐G‐MTX to macrophages within 24 h. b) Macrophage uptake assay: i) free MTX‐FITC uptake by nonactivated macrophages, ii) G‐MTX‐FITC uptake by nonactivated macrophages, iii) free MTX‐FITC uptake by LPS‐activated macrophages, iv) G‐MTX‐FITC uptake by LPS‐activated macrophages. Scale bar: 100 μm. c,d) M1 (CD86, green) and M2 (CD206, red) markers were used for immunofluorescence staining to evaluate the phenotypic changes of macrophages. Scale bar: 100 μm. e–g) ELISA analysis to detect protein expressions of TNF‐α, IL‐6, and IL‐1β in different groups. *n* = 3. Data were presented as mean ± SD. Statistical significance was calculated by one‐way ANOVA, *0.01 < *P* < 0.05, **0.001 < *P* < 0.01, ***P < 0.001.

The safety of biomaterials has always been a concern in practical biomedical applications. In order to determine the biocompatibility of HAMA, raw 264.7 macrophages were cocultured with the extracts of HAMA microspheres. The Calcein AM/PI staining result showed that HAMA had minimal cell toxicity (Figure S5a, Supporting Information). Since the nanoparticles (G‐MTX) will circulate blood through the bone penetrating synovium, a hemolysis test was conducted to examine the safety of the prepared G‐MTX systemically drug delivery preparation. Free MTX produces minor hemolysis at low concentrations (6.25–25 μg mL^−1^) and free MTX causes hemolysis of almost 15% of red blood cells (RBCs) at high concentrations (25–100 μg mL^−1^). On the contrary, G‐MTX hardly produced any hemolysis (≤ 6.5%), with a concentration range of 6.25–100 μg mL^−1^ (Figure S5b, Supporting Information). These results indicated that the peptide dendrimer‐grafted MTX nanoparticles had good blood biocompatibility and provided an important safety basis for further in vivo studies. CCK‐8 assays were performed to assess the cytotoxicity of G3KDSP and G‐MTX on raw 264.7 macrophages. The cells treated with G3KDSP maintained good viability at concentrations ranging from 0.1 to 50 μg mL^−1^, demonstrating the absence of cytotoxic effects and the good biocompatibility of G3KDSP as a drug carrier material (**Figure** **3**a). In addition, G‐MTX exhibited lower cytotoxic effects with increasing concentration compared to free MTX. These results suggested that G‐MTX had the ability to become a nanodrug carrier system to reduce the cytotoxic effects of MTX and deliver to RA targets.

Since macrophages are inflammatory mediators (tumor necrosis factor α (TNF‐α), interleukin 6 (IL‐6), interleukin 10 (IL‐10), interleukin 1β (IL‐1β)), the basic source of secretion, and a large number of them exist in inflammatory sites. By monitoring the uptake of nanoparticles in activated macrophages, it is crucial to evaluate the effectiveness of nanoparticles in treating RA. Both nonactivated (M0) and lipopolysaccharide‐activated macrophages (M1) were used in the macrophage uptake test. There were no discernible variations between nonactivated and activated macrophage uptakes of free MTX that were FITC marked. For FITC‐marked G‐MTX, activated macrophages showed strong green fluorescence; in contrast, for normal macrophages (without lipopolysaccharide (LPS) stimulation), faint green fluorescence was seen in some cells, indicating that the uptakes of G‐MTX by LPS‐activated macrophages were significantly superior to that of unactivated macrophages (Figure 3b). The target cell's ability to be absorbed by the nanoparticles could be increased, and the delivery effect could be improved. To explore whether the nanoparticles grafted with MTX have a synergistic effect on the treatment of RA, in RA joints, proinflammatory factors are secreted by M1‐type macrophages while anti‐inflammatory factors are produced by M2‐type macrophages. Immunofluorescence photos of macrophages with different treatments showed that the number of CD206 (M2 marker) cells was significantly higher and CD86 (M1 marker) cells were lower in the G‐MTX treatment group than MTX‐ and peptide dendrimer‐treated groups (Figure 3c,d). Subsequently, we detected the expression level of macrophage‐related inflammatory cytokines through enzyme linked immunosorbent assay (ELISA) analysis. The G‐MTX showed best performance on reducing TNF‐*α*, IL‐6, and IL‐1*β* secretion than other groups (Figure 3e–g), suggesting that the G‐MTX had a synergistic effect on the treatment of RA compared with the single use of MTX and peptide dendrimer.

**Figure 4:**
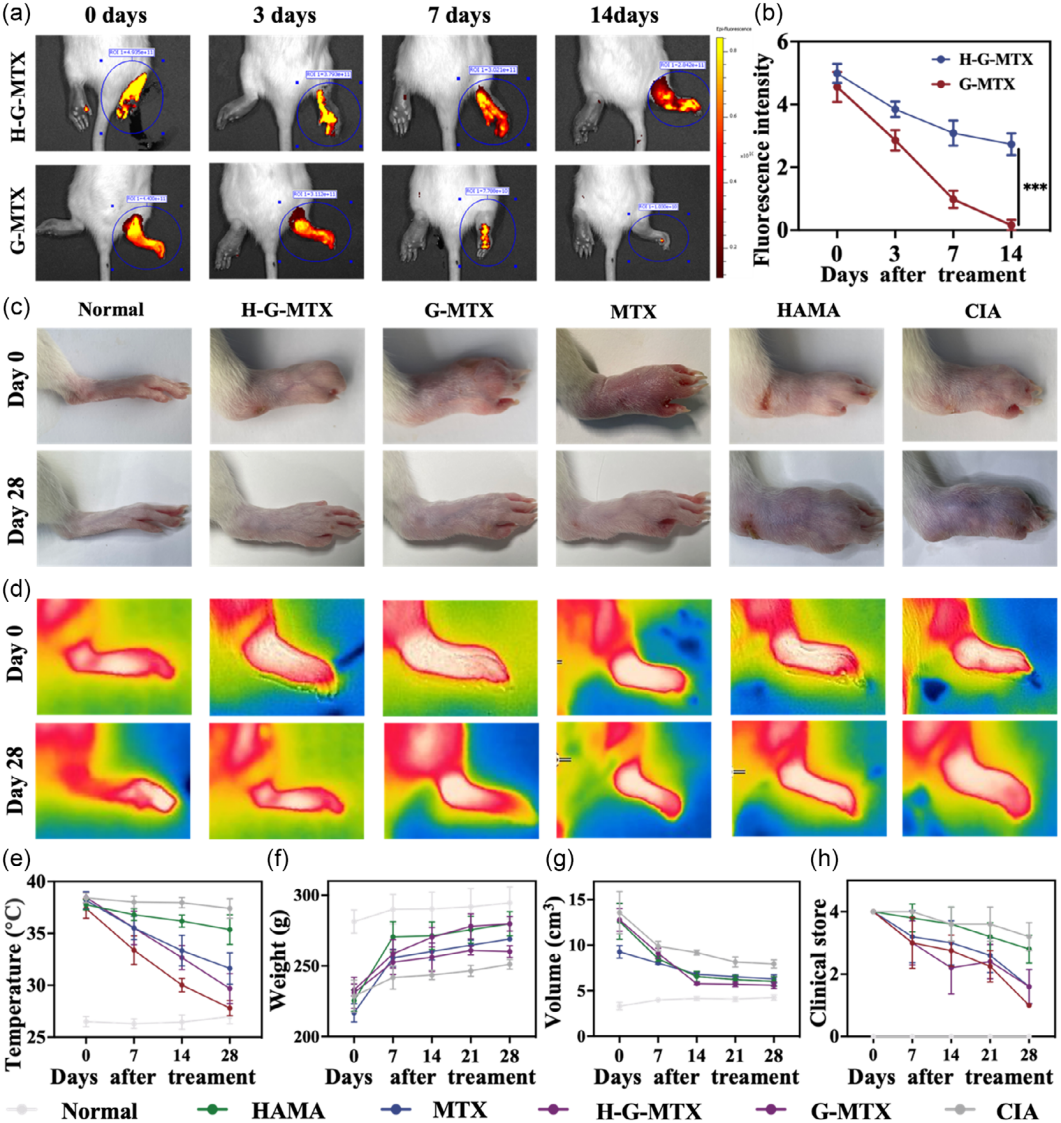
a) The IVIS images of AF750 NHS‐labelled nanoparticles and HAMA package AF750 NHS‐labeled nanoparticles (right ankle joint) at different time points over 14 days. b) The relative fluorescence intensity of each time point. c,d) Photographs of paws and thermal imaging pictures of RA rats in the normal control group, negative control group, and 4 treatment groups before and after 28 days of treatment. e–g) Paw volume, mouse body weight, clinical score, and paw temperature of RA rats before and after 28 days of treatment in normal control group, negative control group, and four treatment groups. *n* = 5. Data were presented as mean ± SD.

Additionally, long‐term drug retention in the articular cavity is crucial for the therapeutic effect of intraarticular drug delivery. Although nanoparticles can effectively improve the delivery efficiency of MTX; when exposed to body fluids, they are easily decomposed and cleared by immune cells. Thus, microspheres are an effective methodology for stabilizing nanoparticles and protecting them from immune clearance. AF750 NHS Ester‐marked G‐MTX encapsulated with HAMA microspheres was injected into the joint cavity of rats, and fluorescence imaging was performed for three consecutive weeks (**Figure** **4**a,b). G‐MTX marked with AF750 NHS Ester was used as control. It was evident that within 7 days, the fluorescence intensity of the G‐MTX treatment group significantly dropped, and no fluorescence signal was detected at 14 days, indicating that the retention ability of G‐MTX in the articular cavity was weak. In contrast, the resistance time of G‐MTX encapsulated in microspheres in vivo could be longer. The result indicated that microspheres could ensure long‐term drug retention in the joint cavity.

Moreover, we developed a collagen‐induced arthritis (CIA) rat model with clinical signs comparable to those of human RA in order to assess the therapeutic efficacy of H‐G‐MTX in vivo. RA was fully established when the hind paws of the experimental rats showed severe swelling and erythema 28 days after immunization. Six groups with various treatment options were created at random from the rats (Normal, CIA, HAMA, MTX, G‐MTX, and H‐G‐MTX). Following four weeks of therapy, the erythema, swelling, and inflammation of rats in the H‐G‐MTX treatment group were ameliorated. Significant relief of paw temperature associated with inflammation was also observed in the H‐G‐MTX treatment group (Figure 4c,d). Compared with other groups, the H‐G‐MTX treatment group showed a significant decrease in the clinical score and paw volume and a consistent increase in body weight (Figure 4f–h). All the above results showed excellent therapeutic effects of H‐G‐MTX, which might be attributed to the prolonged resistance time of the drug in the joint cavity by loading in microspheres and the sustained release of G‐MTX in the acidic microenvironment. Furthermore, the peptide dendrimers in the inflamed joint amplified the efficacy and reduced the toxicity of MTX.

**Figure 5:**
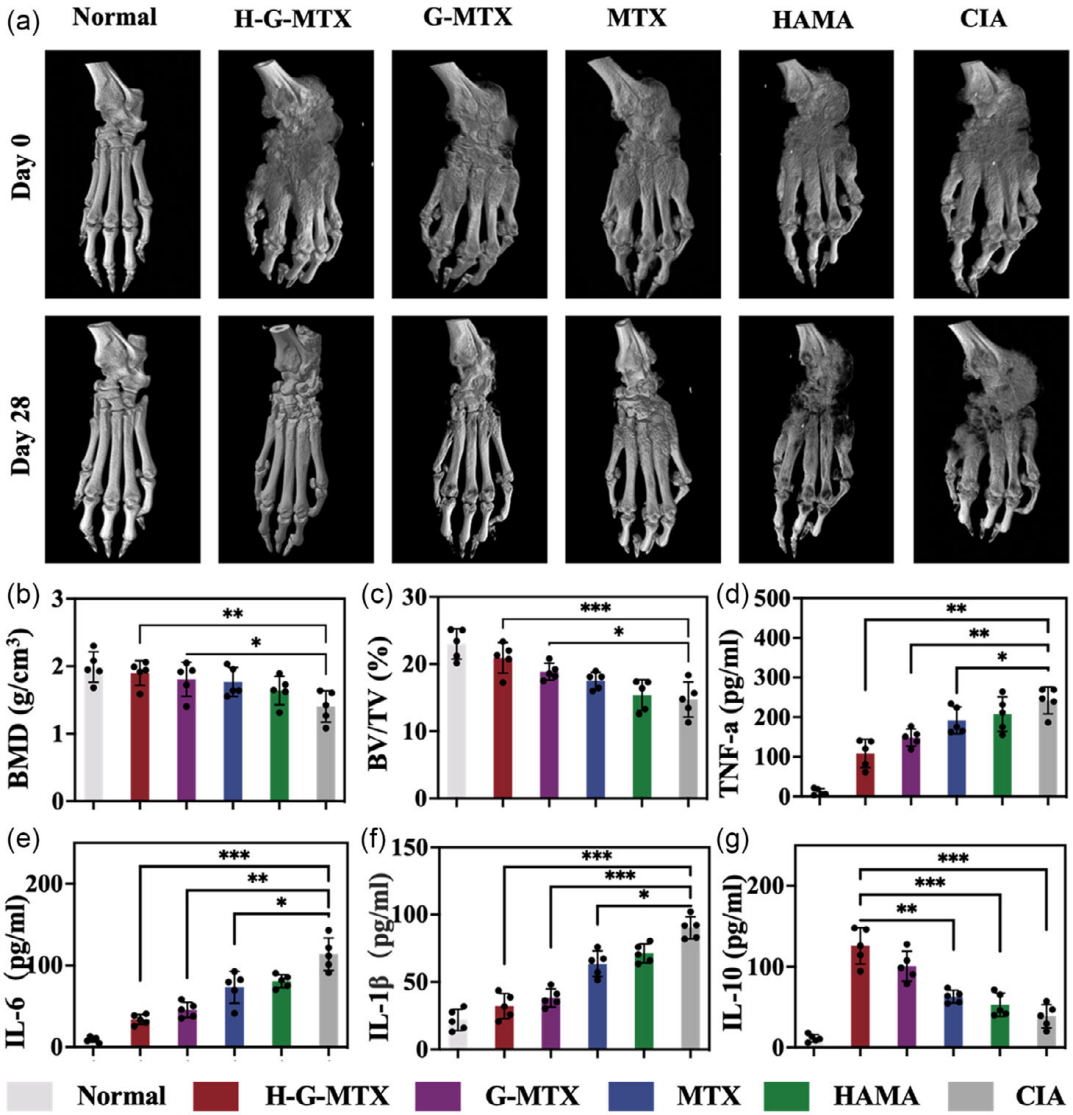
a) X‐Ray images of hind paws and ankle among the different treatment groups. b,c) The quantitative analysis of BV/TV and BMD in six groups 28 days post‐treatment. d–g) TNF‐α, IL‐6, IL‐1β, and IL‐10 analyzed by ELISA. *n* = 5. Data were presented as mean ± SD. Statistical significance was calculated by one‐way ANOVA, *0.01 < *P* < 0.05, **0.001 < *P* < 0.01, ***P < 0.001.

We performed micro‐computed tomography (CT) to evaluate the bone and cartilage erosion of CIA rats. Compared with the CIA and HAMA groups, the bone erosion in both the MTX and G‐MTX groups improved to some extent, with the most significant improvement in the H‐G‐MTX group (**Figure** **5**a). The H‐G‐MTX treatment group showed a significant increase in bone mineral density (BMD) and relative bone volume fraction (BV/TV) (Figure 5b,c). The results demonstrated that H‐G‐MTX could effectively improve bone erosion induced by RA.

**Figure 6:**
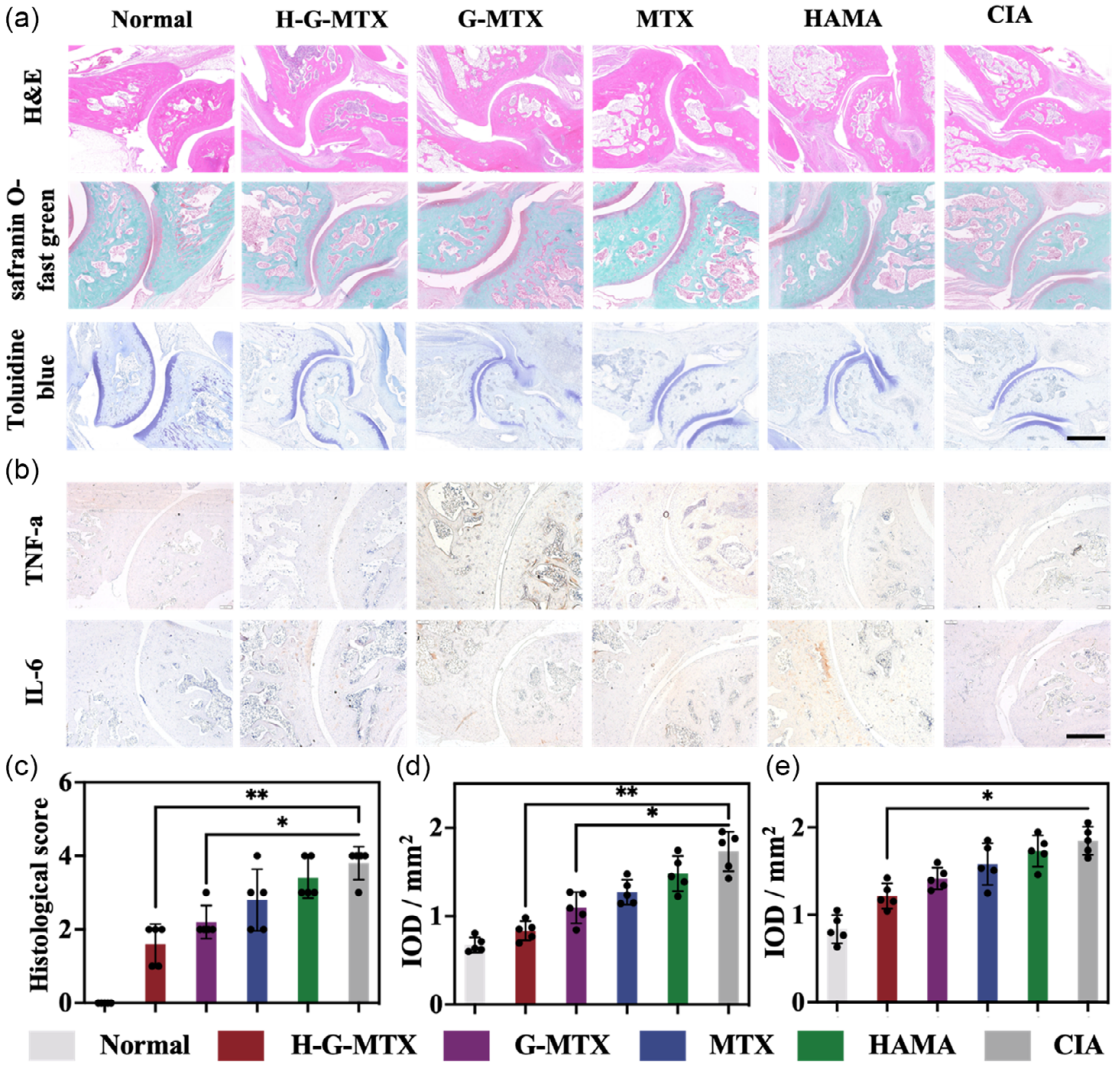
Histological analysis of animal experiments in RA. a) Tissue sections were stained with H&E, toluidine blue (TOL B), safranin O (SAF O). b) Representative immunohistochemical images showing TNF‐α and IL‐6 expressions of synovial in the ankle joints. Scale bar: 1000 μm. c) Histological scores of RA rats for each group. d–e) Quantitative immunohistochemical results for TNF‐a and IL‐6 expressed as mean OD values. *n* = 5. Data were presented as mean ± SD. Statistical significance was calculated by one‐way ANOVA, *0.01 < *P* < 0.05, **0.001 < *P* < 0.01, ***P < 0.001.

H‐G‐MTX considerably reduced proinflammatory factors while dramatically raising anti‐inflammatory factors when compared to CIA controls, according to quantitative examination of the expression levels of inflammatory components in peripheral blood as determined by ELISA (Figure 5d–g). The anti‐inflammatory properties of H‐G‐MTX were then further examined at the in vivo histology level. Normal controls had smooth cartilage surfaces of the ankle and knee joints without soft tissue swelling and uniform joint spaces. The negative control group showed significant joint soft tissue swelling and bone swelling. In contrast, the H‐G‐MTX treatment group showed more significant improvement in cartilage loss and soft tissue swelling in the knee and ankle joints compared to free MTX and G‐MTX. (**Figure** **6**a,c and Figure S6, Supporting Information). These results confirmed that H‐G‐MTX significantly inhibited proinflammatory factors, attenuated cartilage loss due to inflammatory response, and, increased chondrocyte density. We also used immunohistochemical labeling and the ImageJ program to quantify the infiltration of TNF‐ and IL‐6 in the synovium of RA patients (Figure 6b). Quantitative analysis of immunohistochemical staining showed that the expression levels of TNF‐α and IL‐1β were more significantly reduced in the H‐G‐MTX group (Figure 6d,e and S7, Supporting Information). In addition, H‐G‐MTX did not produce significant histological damage based on liver and kidney function and histological analysis of major organs, confirming the biocompatibility of H‐G‐MTX (Figure S8 and S9, Supporting Information). Overall, H‐G‐MTX is biocompatible, reduces inflammatory response, protects cartilage, and improves bone erosion. This graded hierarchical system has broad application prospects.

## 3 Conclusion

In conclusion, we presented a locally injectable hierarchical delivery system with HA microspheres and MTX‐conjugated nanoparticles for facilitated RA treatment. Dendrimer chemical grafting of MTX could improve the water solubility and cellular uptake efficiency of MTX and reduce toxicity. By loading G‐MTX into HAMA microspheres, the resultant hierarchical H‐G‐MTX microspheres exhibited good biocompatibility and could effectively promote the phenotypic transformation of macrophages from M1‐type to M2‐type. Moreover, the H‐G‐MTX microspheres displayed longer retention time in the CIA joints and significantly reduced inflammation, protected cartilage, and promoted bone density. These results indicated that the proposed hierarchical delivery system had potential clinical value in the treatment of RA and other diseases.

## 4 Experimental Section

4.1

4.1.1

##### Materials

MTX can be purchased from Aladdin Co., Ltd. (Shanghai, China). HA (Mw=10 kDa) was purchased from Bloomage Biotechnology Corporation Limited. NHS, 1‐(3‐dimethylaminopropyl)‐3‐ethylcarbodiimide hydrochloride (EDC), DSP, DMSO, DMF, Alexa Fluor 750 NHS Ester, DAPI, FITC, the Cell counting kit‐8 (CCK8), the calcein AM, and propidium iodide (PI) staining kit were obtained from Keygen BioTECH Co., Ltd. (Nanjing, China). PBS, Dulbecco's‐modified Eagle medium, and fetal bovine serum were purchased from United States Origin, Gibco. Immunization‐grade bovine type II collagen solution (2 mg mL^−1^, Chondrex), complete Freund's Adjust (4 mg mL^−1^, Chondrex), and isoflurane (RWD Life Science, China) were obtained from companies.

##### Cells and Animals

Raw 264.7 macrophages, and 8‐week standard deviation (SD) female rats, weighing 200‐300 g, were purchased from Shanghai Sibao Bikai Experimental Animal Co., Ltd. (Shanghai, China). All animal experiments were conducted in a pathogen‐free environment and in accordance with the guidelines for the care and use of experimental animals approved by the Ethics Committee. The animals were placed in standard stainless‐steel cages at 23 ± 0.5 °C. Before the experiment, the animals adapted to the environment for 1 week and provided standard diet and water throughout the study.

##### Synthesis and Characterization of G3K

G3K was synthesized using a divergent technique in accordance with a prior publication.^[^ ^43^, ^44^, ^45^, ^46^ ^]^ The synthesis of G3K was verified using ^1^H NMR (Bruker, Germany, 400 MHz) spectra.

##### Synthesis of HAMA

Briefly, HA (Mw:10 kDa ≈1.0 MDa, Bloomage Biotechnology) (4 g) was dissolved in PBS (Invi‐ nitrogen) (200 mL) at room temperature using a magnetic stirrer. To the HA solution, 15 mL of methacrylic anhydride (Sigma) was slowly added drop wise, stirred vigorously at 0 °C for 24 h, and the pH was conditioned with 1 m NaOH (Aladdin) solution and maintained between 8 and 9. The solution was then dialyzed with a 12–14 kDa membrane for 1 week to remove unreacted methacrylic anhydride. Finally, the dialysis‐completed HAMA solution was lyophilized.

##### Synthesis of H‐G‐MTX

200 mg of G3KDSP, 73.12 g of MTX, 22.24 g of NHS, and 37.04 × g of EDC were dissolved in DMSO for overnight reaction. The reaction products were placed in a dialysis belt with a molecular weight of 1000 and dialyzed in DMF for 2 days and the solution was replaced every 4 h. The reaction products were then dialyzed in deionized water for 2 days and the solution was replaced every 4 h and finally freeze dried to obtain drug‐loaded nanoparticles. The nanoparticles were dissolved in a 5% HAMA solution containing 1% HMPP. Microspheres were prepared by a microfluidic device and irradiated under ultraviolet to form the crosslinked microspheres.

##### Characterization of Nanoparticles and Microspheres

The grafting of peptide dendrimer and MTX at different proportions was characterized by ^1^H NMR. Using dynamic light scattering (DLS), the particle size and zeta potential were determined (DLS). Transmission electron microscopy was used to study nanoparticle morphology. The ultraviolet spectrophotometer was used to measure the grafting rate, drug loading, and encapsulation rate of nanoparticles respectively. The encapsulation rate of nanoparticles was calculated using the following formula: EE (%) = weight of drug grafted on peptide dendrimer/weight of total drug × 100%. The drug loading rate of the nanoparticles was determined using the following equation: DL (%) = weight of drug grafted in peptide dendrimer/weight of total nanoparticles × 100%. The grafting rate of the nanoparticles was measured using the equation below: GR (%) = molar mass of drug grafted on peptide dendrimer /molar mass of total nanoparticles × 100%. With the use of an optical microscope, the microspheres’ morphology was examined, and Image J software was used to determine their diameter. SEM was used to examine the porous architecture of the freeze‐dried microspheres. The nanoparticles dyed with AF750 NHS Ester were encapsulated in the microspheres and we used Thunder to photograph the nanoparticles in the microspheres. Similarly, the UV–vis absorption spectroscopy method was used to determine the encapsulation efficiency and drug loading ratio of microspheres by the following formulas: EE (%) = drug weight encapsulated in microspheres/total drug weight × 100%. DL (%) = weight of drug encapsulated in microspheres/weight of total micelles × 100%.

##### In Vitro Degradation Experiment

50 mg of HAMA microspheres were immersed in PBS (pH 7.4, 5 mL) containing 50 U mL^−1^ hyaluronidase in a shaker (37 °C, 80 rpm), and the solution was replaced every 2 days. The remaining weight of the dried microspheres was measured at the indicated time points on 1, 2, 3, 5, 7, 14, and 21 days. The percentage of degradation (DP) of the microspheres was calculated using the following formula. DP (%) = (*W*_0_ − *W*_1_)/*W*_0_ × 100 (*W*_0_ represents the initial dry weight, *W*_1_ represents the dry weight at the specified time point).

##### In Vitro Release Test

H‐G‐MTX, G‐MTX, and free MTX (3 mg) were added in 2–3 mL of PBS (pH5/pH7.4) in dialysis membranes (Mw = 14kDa). The dialysis membranes were immersed in 100mL PBS. At specific time points (1, 2, 4, 6, 8, 10, 12, 14, 16, 18, 20 d), 2 mL of sample was aspirated, and fresh PBS was added. The concentration of drug released was measured by UV–vis absorption spectrometry.

##### Cell Viability Test

Raw 264.7 macrophages were used to evaluate the biocompatibility of HAMA. Macrophages were cultured overnight in 12‐well plates (Thermo, USA). Then, the experimental group received an additional HAMA infusion, and 72 h of incubation were completed. Calcein AM/PI staining was used to determine cell viability after 24, 48, and 72 h.

##### Hemolysis Test

The toxicity of nanoparticles was assessed using an in vitro hemolysis technique and rat RBCs were collected to assess the hemolytic capability of MTX/G‐MTX. The RBCs were centrifuged at 4 s the hemolytic capabiltes and suspended in 10 mL saline. 500 μL of RBC suspension was incubated in 500 μL of different concentrations (6.25, 12.5, 25, 50 μg mL^−1^) of MTX/G‐MTX solution and PBS and deionized water. The sample was centrifuged at 2500 g for 10 min after being incubated at 37 °C for 3 h. On a 96‐well plate, 150 μL of the supernatant was collected and analyzed for hemoglobin content using a 540 nm microplate reader. Hemolysis rate (%) = (sample‐non reactive control)/(positive control‐non reactive control) × 100%.

##### Cell Uptake Test

In a 12‐well plate, LPS‐activated macrophages and nonactivated macrophages were seeded and cultured overnight. Afterward, FITC‐labelled MTX and G‐MTX were cocultured with macrophages for 4 h. The uptake of MTX and G‐MTX by activated and inactivated macrophages was observed using laser scanning confocal microscopy (CLSM).

##### Cytotoxicity of G‐MTX

To assess the cytotoxicity of G3KDSP and G‐MTX on raw 264.7 macrophages, in a 96‐well plate (2.0 × 104 cells well^−1^), macrophages were seeded and cultured for 24 h. Then, various G3KDSP, MTX, and G‐MTX solution concentrations (100, 50, 25, 10, 1, 0.1 μg mL^−1^) were added, and the mixture was incubated for 24 h. The supernatant was then drained, 100 μL of media containing 10% CCK‐8 solution was added to each well, and the mixture was incubated at 37 °C for 1 h. A microplate reader was used to measure the optical density (OD) at 540 nm.

##### Macrophage Phenotype Transition and Inflammatory Factor Determination

The raw 264.7 macrophages were seeded in a 12‐well plate with coverslips and activated with LPS (1 μg mL^−1^) for 48 h. The LPS‐activated macrophages were incubated in a medium with the addition of PBS, MTX, G3KDSP, and G‐MTX (equivalent to MTX 50 μg mL^−1^) for 24 h, followed by fixing with 4% paraformaldehyde. Cells were then incubated with primary antibodies against CD68, CD206, or CD86 overnight at 4 °C, followed by 30 min incubation with the respective fluorescently labeled secondary antibodies. Cell nuclei were stained with DAPI and samples were observed and imaged with a CLSM. In addition, the unactivated macrophages were incubated in a medium added with PBS for 24 h. TNF‐α, IL‐6, and IL‐1β levels in the supernatants of the five groups were then evaluated using an Elisa kit.

##### Animal Model Induction and Treatment

All in vivo experiments were performed according to the Ethics Committee of Drum Tower Hospital and the approval number issued by the Laboratory Animal Welfare Ethics Committee of Drum Tower Hospital was 2021AE01008. The CIA rat model was established by tail vein injection of an emulsion made from a homogenizer with a small blade (5 mm diameter) to emulsify IFA (4 mg mL^−1^) with collagen solution (2 mg mL^−1^). All rats had free access to sterile food and water and were monitored daily for the progression of arthritis during disease progression. On day 0, rats were injected with 100 μl of PBS, MTX, G‐MTX, H‐G‐MTX (equivalent to MTX 5 mg kg^−1^) and HAMA into the hind paw joints, respectively. The progression of arthritis in rats was monitored daily.

##### Evaluation of Residence Time In Vivo

50 μl of nanoparticles and microspheres loaded with nanoparticles were labeled AF750 NHS ester and were injected into the right ankle joint of rats and photographed on 1, 3, 7, 14, and 21 days using an IVIS spectroscopy system (Xenogen, Hopkinton, MA, USA).

##### Analysis of Serum Markers and Evaluation of Liver and Kidney Function

On the 28th day after administration, blood and organs were collected from each group. According to the standard protocol, the ELISA kit was used to determine the expression level of the proinflammatory cytokine (TNF‐α, IL‐6, IL‐10, and IL‐1β). The levels of serum aspartate aminotransferase, alanine aminotransferase, alkaline phosphatase, creatinine, blood urea nitrogen, and uric acid were measured by the automatic biochemical analyzer.

##### Microcomputer Tomography Analysis of Ankle Joint

On the 29th day after administration, six groups of rats (*n* = 5) were euthanized. 4% paraformaldehyde was used for 24 h for right ankle joint fixation and high‐resolution microcomputer tomography (Micro CT, Siemens Healthcare, Berlin and Munich Germany) was used to assess the bone density and BV fraction of the ankle joint. Micro‐CT scanning parameters were set as voltage 60 kV and current 134 μA. Resolution was 50 μm. The low density was 1.26 g cm^−3^, the low density ct value was 1469HU, the high density was 1.9 g cm^−3^, and the high density ct value was 4240HU. For visualization and data analysis, 3D reconstructions were performed.

##### Histological Analysis of Joints

Neutral calcium EDTA decalcification solution (14%) was used to completely decalcify the joints in the ankle and knee. The decalcified ankle joint was paraffin embedded and divided longitudinally along its middle axis. In 5 mm‐thin slices, H & E, safranine fixation green, and toluidine blue were performed in accordance with accepted methods for histological evaluation. The staining score was performed according to the following scale: 0 = no inflammatory cell infiltration, no synovial hyperplasia, no cartilage, and bone destruction; 1 = slight cell infiltration, inflammation with mild hyperplasia of synovium, mild destruction of cartilage and bone; 2 = moderate inflammatory cell infiltration, moderate synovial hyperplasia, moderate cartilage, and bone destruction; 3 = inflammatory cell infiltration, synovial hyperplasia, cartilage, and bone destruction were further aggravated; 4 = a large number of inflammatory cell infiltration, severe synovial hyperplasia, and severe cartilage destruction with a large area of bone loss.

##### Statistical Analysis

All data were expressed as mean ± SD. The significant difference between groups was analyzed by one‐way ANOVA in the GraphPad Prism 6.0 (GraphPad Software, La Jolla, California, USA). Statistical significance was defined as *p < 0.05, **p < 0.01, ***p < 0.001.

## Conflict of Interest

The authors declare no conflict of interest.

## Author Contributions

The author contribution is as follows. Y.L. and H.Z. contributed equally to this work. Y.L. contributed to data curation and writing the original draft. H.Z contributed to writing the review and editing. R.L. took care of validation. Y.Z. took care of conceptualization, methodology, and supervision. L.S. took care of conceptualization, methodology, and supervision.

## Supporting information

Supplementary Material

## Data Availability

Research data are not shared.
