# Correction to “Hierarchical Microcarriers Loaded with Peptide Dendrimer‐Grafted Methotrexate for Rheumatoid Arthritis Treatment”

**DOI:** 10.1002/smsc.202400614

**Published:** 2025-01-08

**Authors:** Yang Li, Haofang Zhu, Rui Liu, Yuanjin Zhao, Lingyun Sun

*Small Sci.* **2023**, *4* (1), 2300097, https://doi.org/10.1002/smsc.202300097

1. The images of **Figure** **3**b (iv group) and Figure 3d (Merge line) were wrongly pasted during the assembly of Figure 3. The correct figure is as follow:

**Figure 3:**
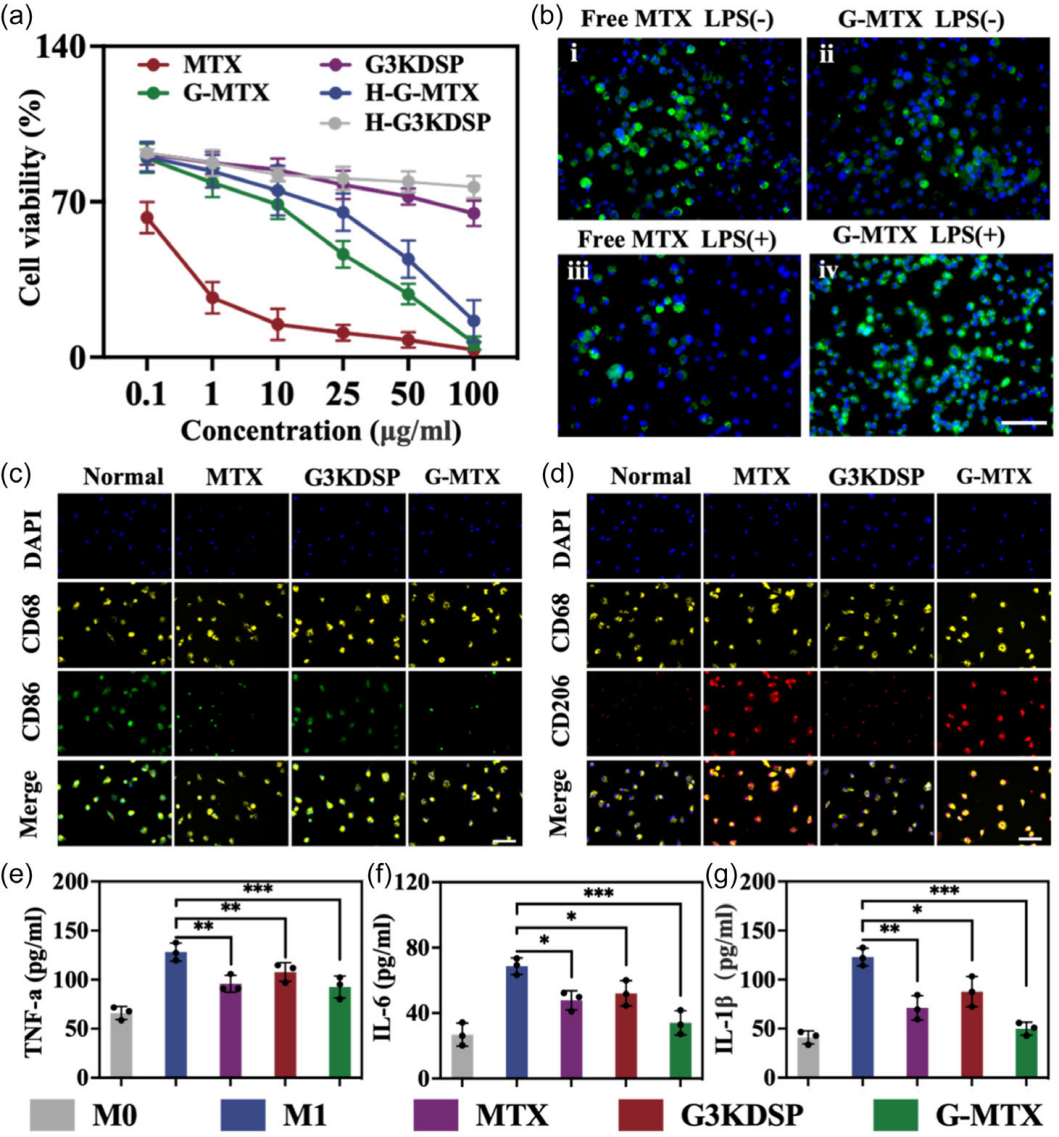
a) The cytotoxicity of G3KDSP, MTX, G‐MTX, H‐G3KDSP or H‐G‐MTX to macrophage within 24 h. b) Macrophage uptake assay: i) Free MTX‐FITC uptake by non‐activated macrophages, ii) G‐MTX‐FITC uptake by non‐activated macrophages, iii) Free MTX‐FITC uptake by LPS activated macrophages, iv) G‐MTX‐FITC uptake by LPS activated macrophages. Scale bar: 100 μm. c,d) M1 (CD86, green) and M2 (CD206, red) markers were used for immunofluorescence staining to evaluate the phenotypic changes of macrophages. Scale bar: 100 μm. e–g) ELISA analysis to detect protein expressions of TNF‐α, IL‐6 and IL‐1β in different groups. *n *= 3. Data were presented as mean ± SD. Statistical significance was calculated by one‐way ANOVA, *0.01 < *P* < 0.05, **0.001 < *P* < 0.01, ***P < 0.001.

2. The image of **Figure** **4**d (second figure of Day 28) was wrongly pasted during the assembly of Figure 4. The correct figure is as follow:

**Figure 4:**
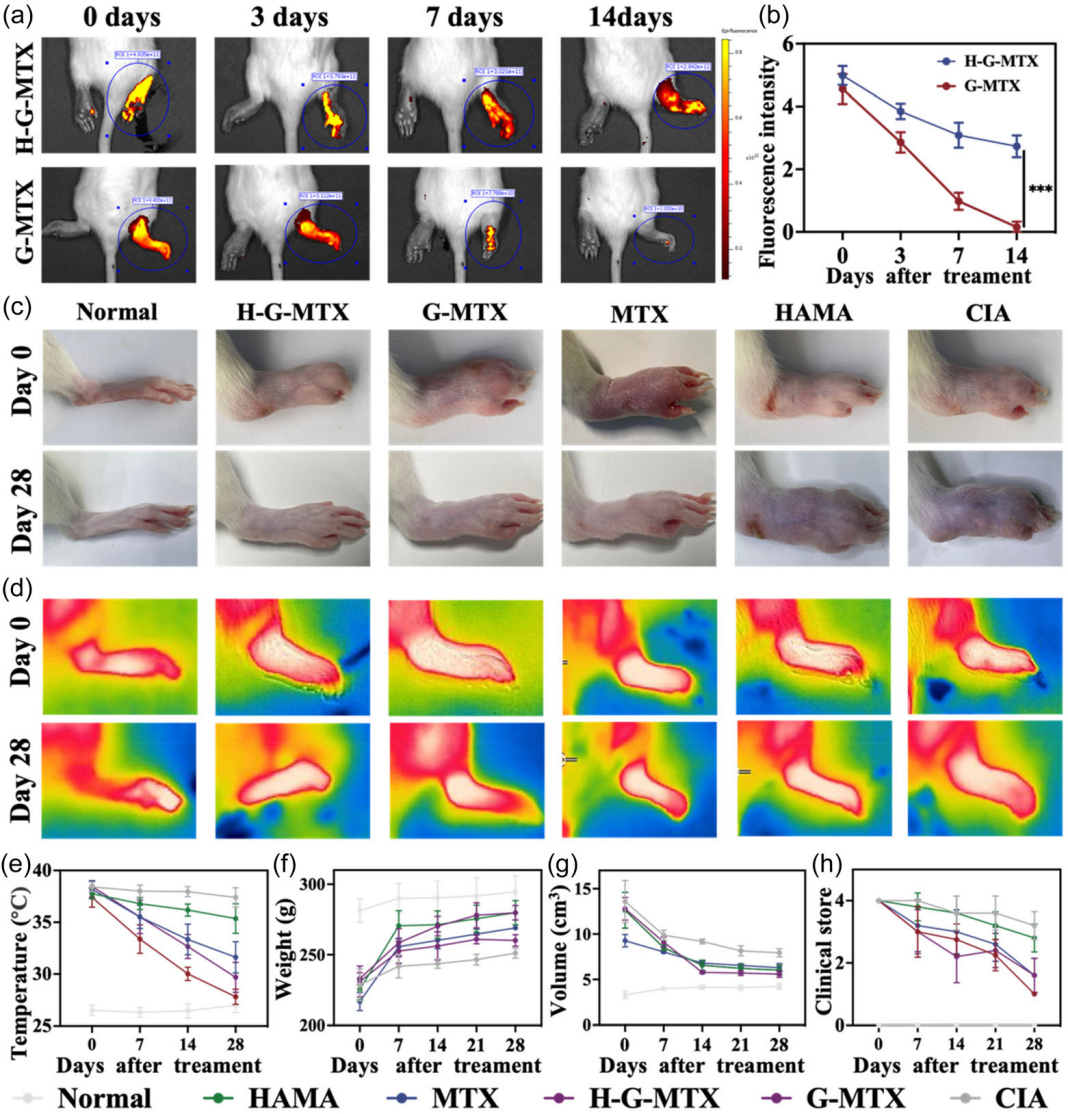
a) The IVIS images of AF750 labelled Nanoparticles and HAMA Packages AF750 labelled Nanoparticles (right ankle joint) at different time points over 14 days. b) The relative fluorescence intensity of each time points. c,d) Photographs of paws and thermal imaging pictures of RA rats in the normal control group, negative control group, and 4 treatment groups before and after 28 days of treatment. e–g) Paw volume, mouse body weight, clinical score and paw temperature of RA rats before and after 28 days of treatment in normal control group, negative control group, and 4 treatment groups. *n* = 5. Data were presented as mean ± SD.

These errors do not affect the results or conclusions of the paper. We sincerely apologize for any confusion this may have caused.

