# Supplementary material for: Hierarchical Microcarriers Loaded with Peptide Dendrimer‐Grafted Methotrexate for Rheumatoid Arthritis Treatment
Source: Small Sci. 2023 Nov 27;4(1):2300097. doi: 10.1002/smsc.202300097 (PMC11935003; doi:10.1002/smsc.202300097)
Supplement: Supplementary file 1 — Supplementary Material [file SMSC-4-2300097-s001.pdf]

Supporting Information

Hierarchical microcarriers loaded with peptide dendrimer grafted methotrexate for rheumatoid arthritis treatment

Yang Li [a,#], Haofang Zhu [a,#], Rui Liu [a], Yuanjin Zhao [a,b,*], Lingyun Sun [a,*]

([#]These authors contributed equally)

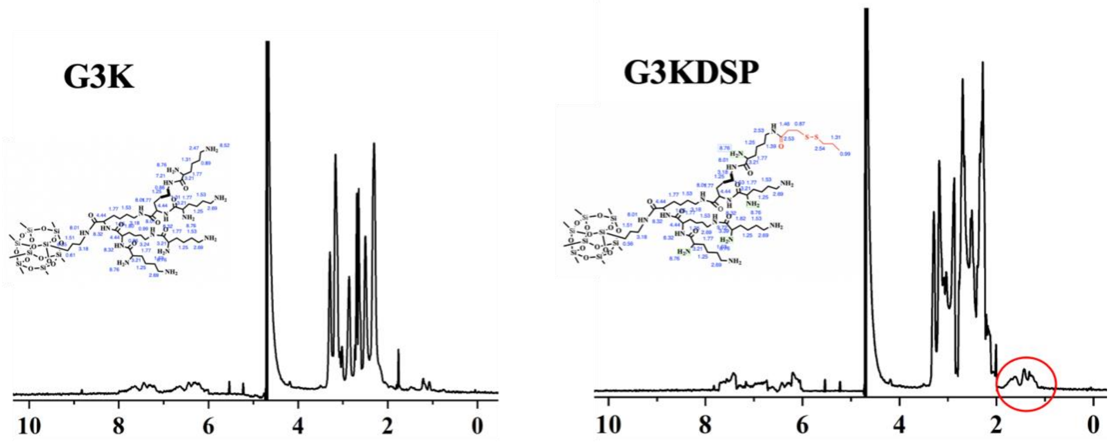

FigureS1: ^{1}H-NMR of G3K and G3KDSP

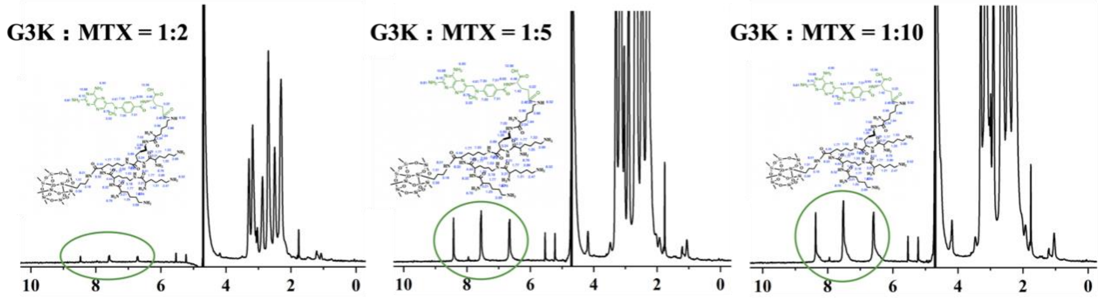

Fig S2. Nuclear magnetic resonance spectroscopy shows the grafting ratio of different proportions of dendrimers grafted with MTX.

a

G3-(NH)64/MTX	1:10	1:5	1:2
GR	12.60%(16.35%)	1.89%(7.81%)	0.67%(3.13%)
DLE	30.63%	13.59%	2.43%
DLC	28.46%	4.26%	1.46%

b

EE（Direct method）	73.6%
Loading capacity	10.52%

Fig S3. a) Drug loading, grafting ratio and encapsulation ratio of the dendrimer grafted with the MTX at 1:2,1:5 and 1:10 ratios. b) The encapsulation efficiency and drug loading of HAMA microspheres encapsulated nanoparticles.

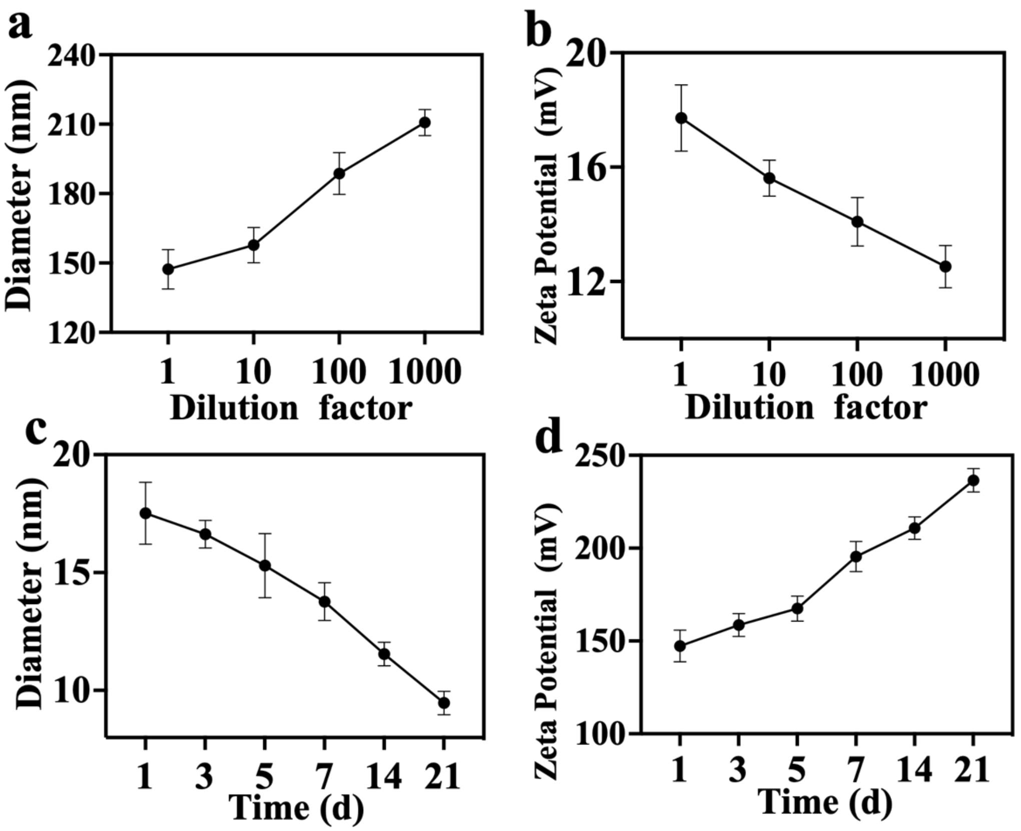

Fig S4. a-b) The particle size potential and change of nanoparticles under the dilution ratio of 1, 10, 100 and 1000. c-d) The particle size potential and change of nanoparticles after 1, 3, 5, 7, 14 and 21days. n = 3. Data were presented as mean ± SD.

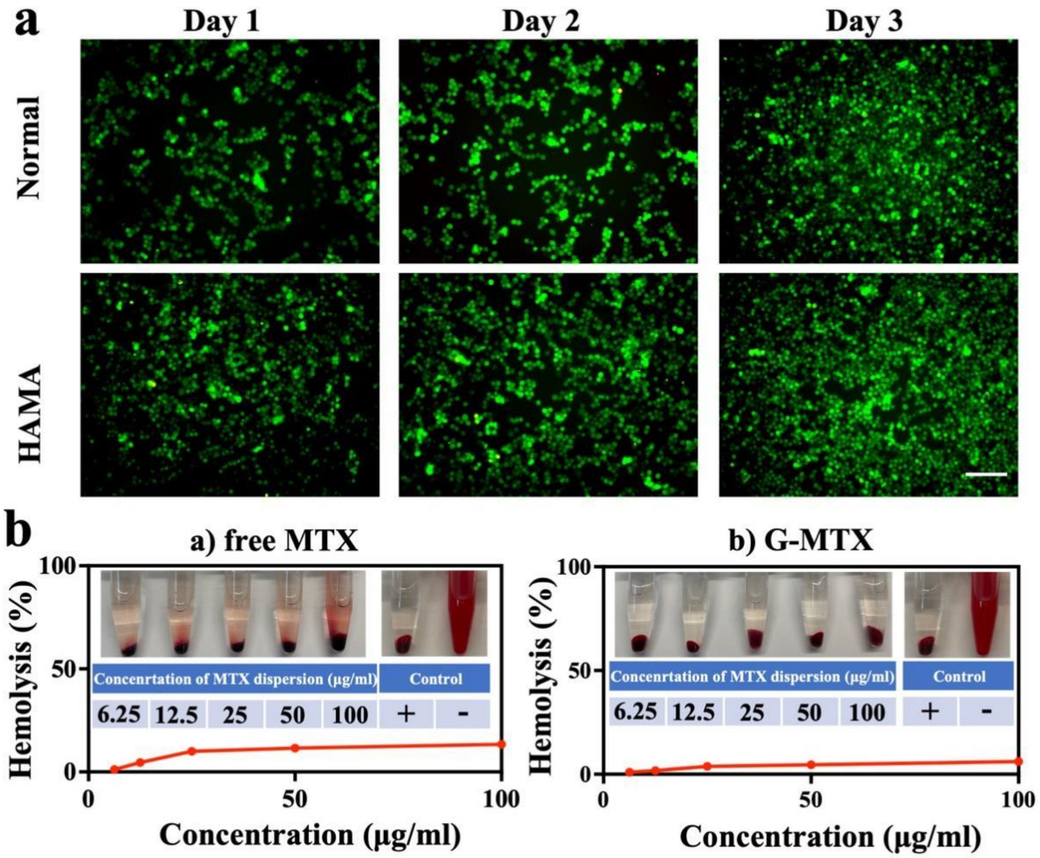

Fig S5. a) Survival and death of normal and HAMA groups in 1, 2 and 3 days. Scale bar: 200 μm. b) Hemocompatibility assay: Percentage hemolysis of RBCs treated with a) free MTX and b) G-MTX.

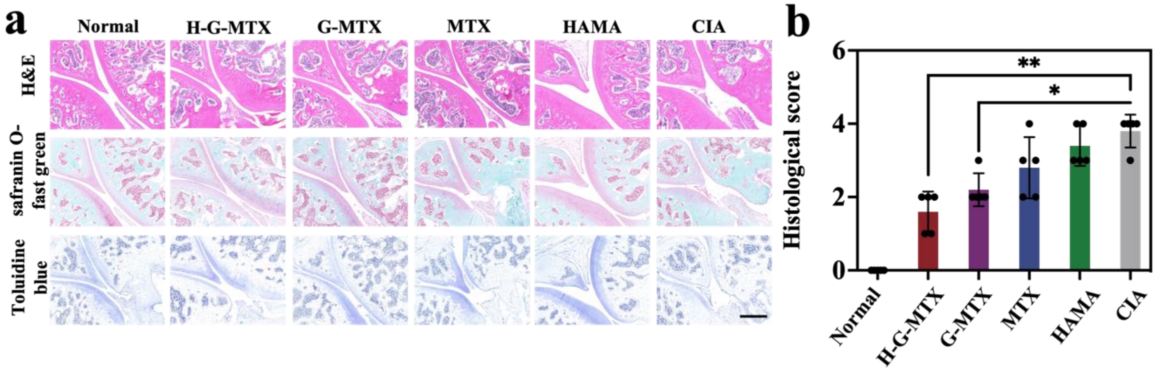

Fig S6. Histological analysis of animal experiments in RA. a) Tissue sections were stained with H&E, toluidine blue (TOL B), safranin O (SAF O). Scale bar: 1000 μm. b) Histological scores of RA rats for each group. n = 5. Data were presented as mean ± SD. Statistical significance was calculated by one-way ANOVA, *0.01 < P < 0.05, **0.001 < P < 0.01, ***P < 0.001.

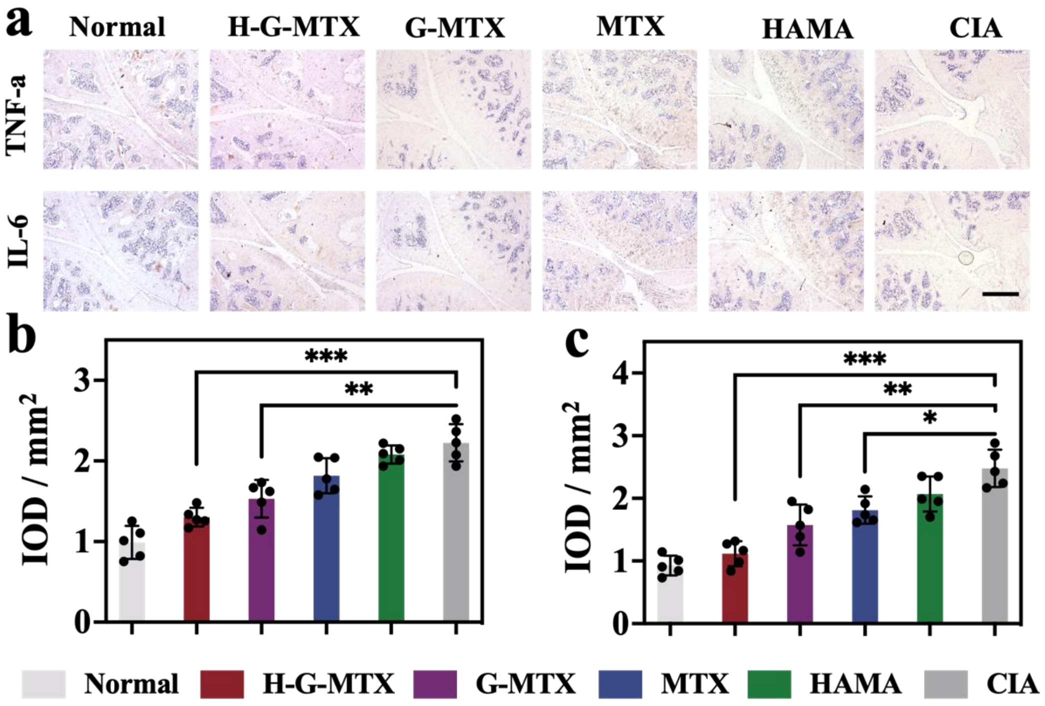

Fig S7. a) Representative immunohistochemical images showing TNF-α and IL-6 expressions of synovial in the knee joints. Scale bar: 1000 μm. b-c) Quantitative immunohistochemical results for TNF-a and IL-6 expressed as mean optical density values. n = 5. Data were presented as mean ± SD. Statistical significance was calculated by one-way ANOVA, *0.01 < P < 0.05, **0.001 < P < 0.01, ***P < 0.001.

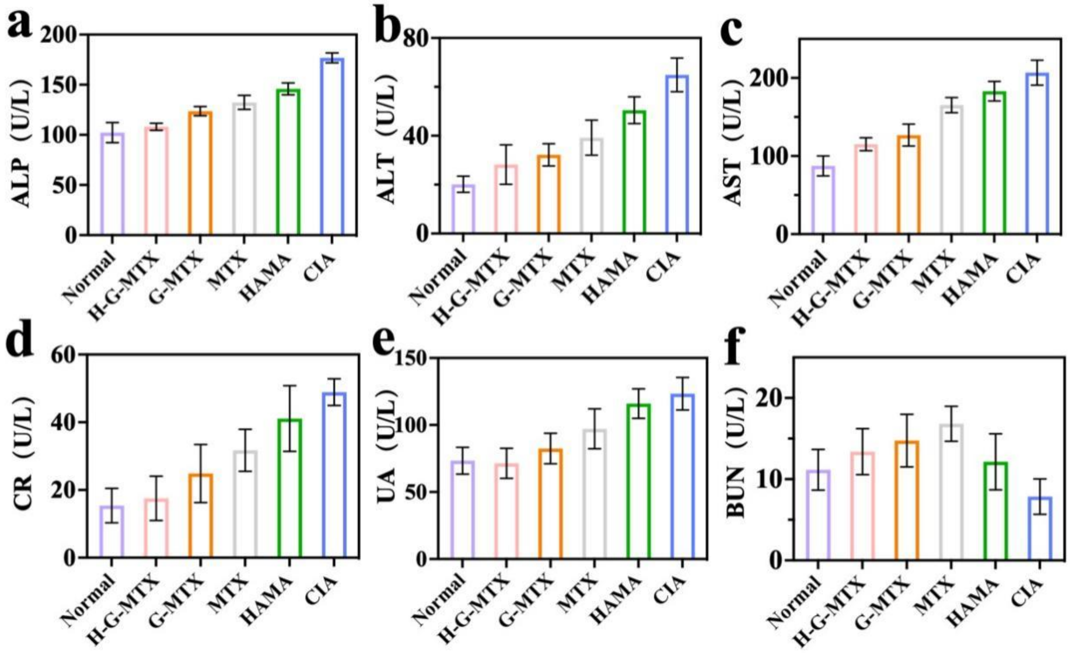

Fig S8. a-f) ALP, ALT, AST, CREA, UA and BUN measured using an automatic biochemical analyzer. n = 5. Data were presented as mean ± SD.

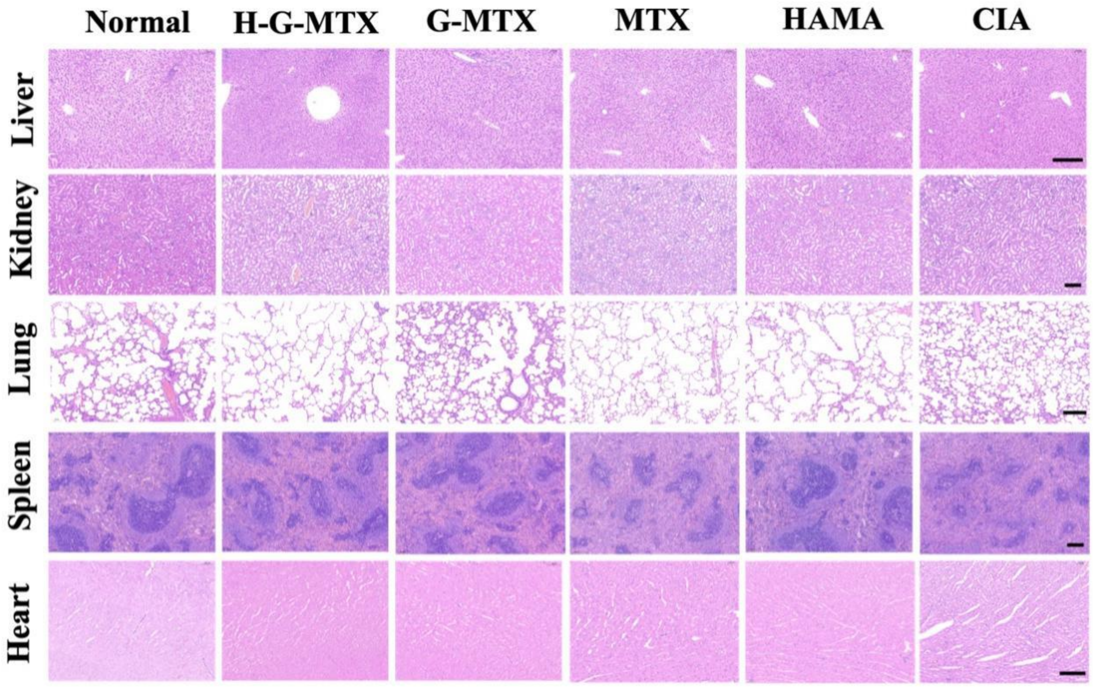

Fig S9. H&E staining of liver, kidney, lung, spleen and heart of rats in different treatment groups. Scale bar: 200 μm.
